# CD155 downregulation synergizes with adriamycin to induce breast cancer cell apoptosis

**DOI:** 10.1007/s10495-018-1473-8

**Published:** 2018-07-23

**Authors:** Jian Gao, Qianqian Zheng, Yue Shao, Wei Wang, Chenghai Zhao

**Affiliations:** 1 Department of Pathophysiology, College of Basic Medical Science, China Medical University, Shenyang, China; 2 Center of Laboratory Technology and Experimental Medicine, China Medical University, Shenyang, China

**Keywords:** CD155, Adriamycin, Breast cancer, Apoptosis

## Abstract

CD155 has been implicated in migration, invasion, proliferation and apoptosis of human cancer cells, and DNA damage response caused by chemotherapeutic agents or reactive oxygen species has been shown to attribute to CD155 induction. Adriamycin (Adr) is one of the most common chemotherapeutic drugs used to treat breast cancer. Here we reported that treatment with Adr upregulated CD155 expression on several in vitro cultured breast cancer cells and in breast cancer cell 4T1 xenografts. We also found that CD155 knockdown or Adr treatment induced apoptosis of in vitro cultured cancer cells and cancer cells in 4T1 xenografts, and a combination of CD155 knockdown with Adr treatment induced more cell death than either of them. Furthermore, we revealed that the combination of CD155 knockdown with Adr treatment suppressed the growth of 4T1 xenografts more significantly than them alone. In summary, our results demonstrate that CD155 downregulation synergizes with Adr to induce breast cancer cell apoptosis, thereby to suppress tumor growth. Our results also suggest that CD155 upregulation may be a mechanism underlying Adr resistance by breast cancer cells.

## Introduction

Due to functioning as the receptor for poliovirus, and harboring domain structures similar to nectins, CD155 is also called PVR [1] and nectin-like protein 5 (necl-5) [2]. CD155 recruits and trans-interacts with nectin three to promote cell migration [2, 3]. CD155 can also enhance cell motility independent on nectin3 but involving integrin αvβ3, Cdc42 and Rac [4]. Moreover, CD155 stimulates cell proliferation by activation of ERK signaling as well as upregulation of cyclins and downregulation of p27 [5]. Through combining with co-stimulatory or co-inhibitory molecules on T or NK cells, CD155 plays implicated roles in tumor immunity [6].

CD155 expression is undetected in most normal tissues, while upregulated in Ras or Src-transformed NIH3T3 cells [5] and several human cancers [7–9]. Moreover, cancer patients have a higher level of soluble isoforms of CD155 in the sera than healthy donors [10]. Overexpression of CD155 is correlated with tumor stage and unfavorable prognosis [8–10]. Mechanistically, CD155 plays a positive role in tumor growth and metastasis. CD155 promotes tumor cell proliferation, invasion and migration [9, 11–13]. Moreover, CD155 expression is associated with tumor VEGF expression and intratumoral vessel density [9]. Recently, our group reported that CD155 inhibited tumor cell apoptosis [14].

CD155 expression can be regulated by several signaling pathways such as Ras, sonic hedgehog (Shh) and TLR4 [5, 15, 16]. Moreover, CD155 expression can be induced by DNA damage. For instance, reactive oxygen species (ROS) and reactive nitrogen species (RNS) stimulate CD155 expression through DNA damage response (DDR) [17, 18]. Similarly, chemotherapeutic agents induce CD155 expression on multiple myeloma cells through DDR [19]. Given the positive role of CD155 in tumor growth, in the present study we asked whether Adriamycin (Adr) treatment [20–22] could upregulate CD155 expression in breast cancer cells, and whether CD155 knockdown could affect Adr-induced breast cancer cell apoptosis.

## Materials and methods

### Cell culture and Adr treatment

MAD-MB-231 (ER^−^PR^−^HER2^−^) and MCF-7 (ER^+^PR^+^HER2^−^) cells were cultured in DMEM (Gibco, USA), and T47D (ER^+^PR^+^HER2^−^) and 4T1 (ER^−^PR^−^HER2^−^) cells were cultured in RPMI1640 (Gibco, USA). The culture media were supplemented with 10% fetal bovine serum (FBS). All cells were cultured in a humidified incubator at 37 °C with 5% CO_2_. For Adr treatment, Adr (Solarbio, Beijing, China) was added into cell cultures at a final concentration of 375 nM for 24 h.

### LentiviralshRNA transfection

CD155 shRNA lentivirus (shCD155) and control shRNA lentivirus (scramble) were constructed by Obio Technology Corp., Ltd (Shanghai, China). The shRNA sequence for CD155 is 5′-CCTAGGCTACATCTTTCTT-3′, and for scramble is 5′-TTCTCCGAACGTGTCACGT-3′. For stable knockdown, shRNAs were cloned into pLKD-CMV-Puro-U6-shRNA vector. MCF-7 and 4T1 cells were transfected with 25 MOI of scramble or shCD155 lentivirus.

### Total RNA extraction and quantitative real-time PCR (qPCR)

Total RNA was isolated from cells using TRIzol™ reagent (TaKaRa, Dalian, China), and reverse-transcribed using M-MLV reverse transcriptase (Promega, Madison, USA). The primers for CD155 are 5′-GCTAGAAGGACTCACTAGACTCAGGAA-3′ (forward) and 5′-GTCGCCTCATCTGTCGTGGAAC-3′ (reverse), and for 18S are 5′-GCAGAATCCACGCCAGTACAAGAT-3′ (forward) and 5′-TCTTCTTCAGTCGCTCCAGGTCTT-3′ (reverse). Quantitative real-time PCR was performed using 2× SYBR Green Mix (Promega, Madison, USA) in Roche Light Cycler 480 PCR Cycler. The PCR program consisted of an initial denaturation step at 95 °C for 5 min, followed by 40 cycles of 95 °C for 15 s and 60 °C for 1 min. Gene expression was calculated relative to 18S.

### Immunofluorescence

Cells were seeded onto adhesion cover slips for 24 h, fixed with 4% paraformaldehyde in PBS for 15 min at 25 °C and permeabilized with 0.1% Triton X-100. Subsequently, cells were blocked with 1% BSA for 30 min and incubated overnight at 4 °C with anti-CD155 antibody (Abcam, 103630, 1:200), followed by incubation with secondary antibody Alexa Fluor 488-conjugated IgG (Life Technologies, Carlsbad, USA, 1:300) for 1 h. DAPI was used for nuclear staining. Cells were mounted on glass slides and visualized using a confocal microscope (FV-1000, Olympus, Japan).

### Western blot

Total protein was extracted from cells using RIPA lysis buffer with 1% PMSF. 40 µg protein was separated on 9% SDS–PAGE gel and transferred on a PVDF membrane. The membrane was blocked with 5% skimmed milk and incubated with anti-CD155 antibody (Abcam, 103630, 1:500) at 4 °C overnight. Then the membrane was washed and incubated with HRP-conjugated secondary antibody (1:5000) for 2 h at room temperature. The specific protein bands were visualized using the enhanced chemiluminescence (ECL) method through the DNR Imaging System. GAPDH was used for normalization. The optical density (OD) of each band was quantified with Image J (Bethesda, MD, USA).

### Cell apoptosis assay by flowcytometry

The Annexin V-PE/7-AAD Apoptosis Kit (559763; BD Biosciences, USA) was used to detect cell apoptosis in a flow cytometer (BD Biosciences, USA), according to the manufacturer’s protocol.

### Xenograft model of breast cancer

Pathogen-free female 8-week BALB/c mice were obtained from the Weitong Lihua (Beijing, China). 1 × 10^4^ 4T1-scramble cells or 4T1-shCD155 cells were injected into the fourth mammary fat pad on day 0. Some mice were treated with Adr (1 mg/kg, i. p.) [23] on day 3, 10, 17, 24, 31. All mice were euthanized on day 35. Tumor volume was calculated by the formula of *V* = ab^2^/2. Animal welfare and experimental procedures were performed according to the Guide for the Care and Use of Laboratory Animals (Ministry of Science and Technology of China, 2006), and were approved by the animal ethics committee of China Medical University.

### Immunohistochemistry and HE staining

The tumor tissues were fixed by 4% paraformaldehyde for 72 h, embedded in paraffin, and sectioned into 4 µm sections. Sections were first stained with hematoxylin and eosin (HE). For immunohistochemistry, sections were incubated with anti-CD155 (Abcam, 103630, 1:200) overnight at 4 °C. Then, sections were incubated with goat anti-rabbit IgG and streptavidin peroxidase (SP) complex at 37 °C for 30 min, and developed with the DAB reagent. The sections were observed under an Olympus BX61 microscope.

### TUNEL staining

Tumor tissue apoptotic cells were determined by the TUNEL Apoptosis Detection Kit S7110 (Millipore, USA) according to the provided protocol. After TUNEL labeling, nucleus was counterstained with DAPI. The TUNNEL positive labeled apoptotic cells were calculated and photographed using an Olympus BX61 fluorescence microscope. The number of apoptotic cells was counted in a total of 1000 cells by Image J software.

### Statistical analysis

All experiments were carried out at least in three replicates. SPSS 13.0 software was used to perform data statistics. Student’s *t* test or one-way ANOVA was used to evaluate the difference. *P* < 0.05 was considered statistically significant.

## Results

### Adr induces CD155 expression in breast cancer cells

**Fig. 1:**
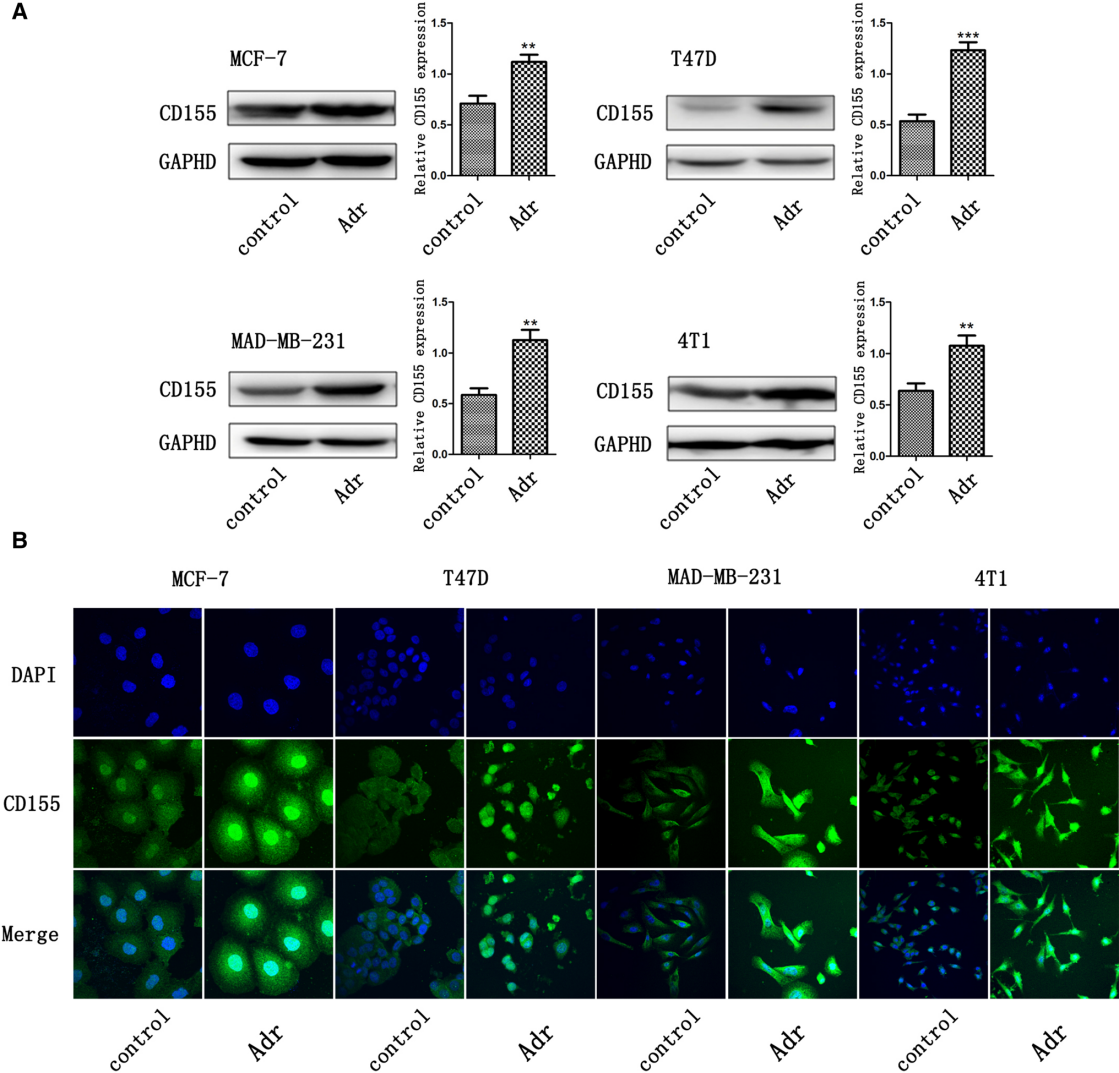
Upregulation of CD155 expression by Adr treatment. Western blot (**a**) and immunofluorescence (**b**) detected CD155 expression in breast cancer cells MCF-7, T47D, MDA-MB-231, and 4T1. **P < 0.01 versus control; ***P < 0.001 versus control. Data are expressed as mean ± SD, and all the experiments were performed three times

Human breast cancer cells MCF-7, T47D and MDA-MB-231 were treated with Adr (375 nM) for 24 h. As shown by Western blot detection, CD155 expression was significantly upregulated in these CD155-treated cancer cells compared with CD155-untreated cells (Fig. 1a). Similar to these human breast cancer cells, mouse breast cancer cell 4T1 also expressed more CD155 after Adr treatment for 24 h (Fig. 1a). CD155 induction by Adr treatment was further confirmed by immunofluorescence staining in these human and mouse breast cancer cells (Fig. 1b).

### CD155 shRNA transfection blocks Adr-induced CD155 expression

**Fig. 2:**
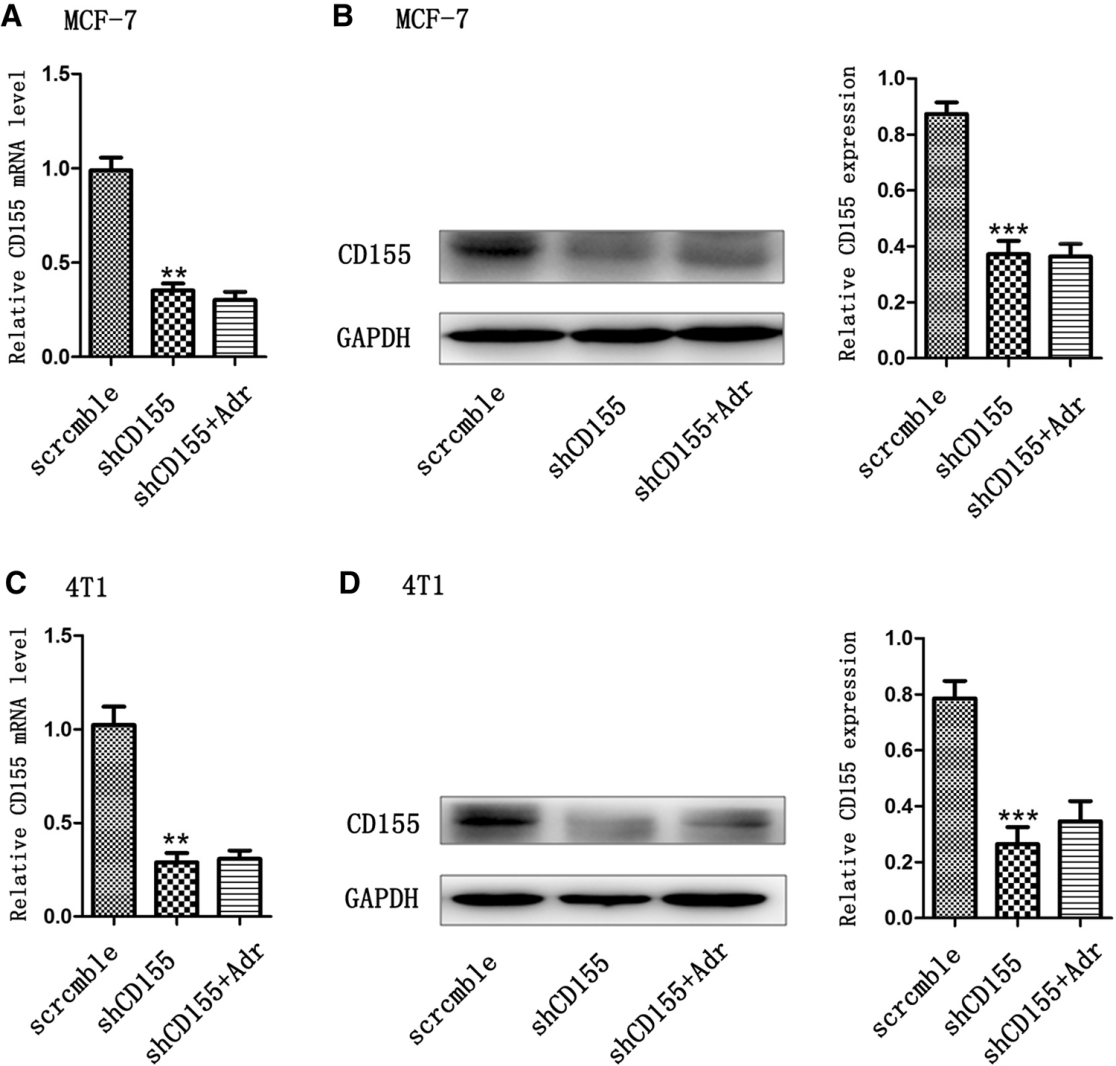
Downregulation of CD155 expression by CD155 shRNA transfection. Real-time PCR (**a, c**) and western blot (**b, d**) detected CD155 mRNA and protein expression in breast cancer cells MCF-7 and 4T1. **P < 0.01 versus scramble; ***P < 0.001 versus scramble. Data are expressed as mean ± SD, and all the experiments were performed three times

MCF-7 cells were transfected with CD155 shRNA lentiviruses. Real-time PCR detection showed that CD155 mRNA level in CD155 shRNA-tranfected cells was significantly lower than that in control cells, indicating that transfection with CD155 shRNA effectively reduced the basal expression of CD155 (Fig. 2a). Furthermore, treatment with Adr failed to increase CD155 mRNA expression in CD155 shRNA-tranfected cells, indicating that transfection with CD155 shRNA also abrogated Adr-induced CD155 expression (Fig. 2a). Subsequent Western blot detection confirmed that transfection with CD155 shRNA suppressed basal and Adr-induced CD155 expression in protein level (Fig. 2b). Similarly, transfection of 4T1 cells with CD155 shRNA inhibited basal and Adr-induced CD155 expression in both mRNA and protein levels (Fig. 2c, d).

### CD155 downregulation synergizes with Adr to induce breast cancer cell apoptosis in vitro

**Fig. 3:**
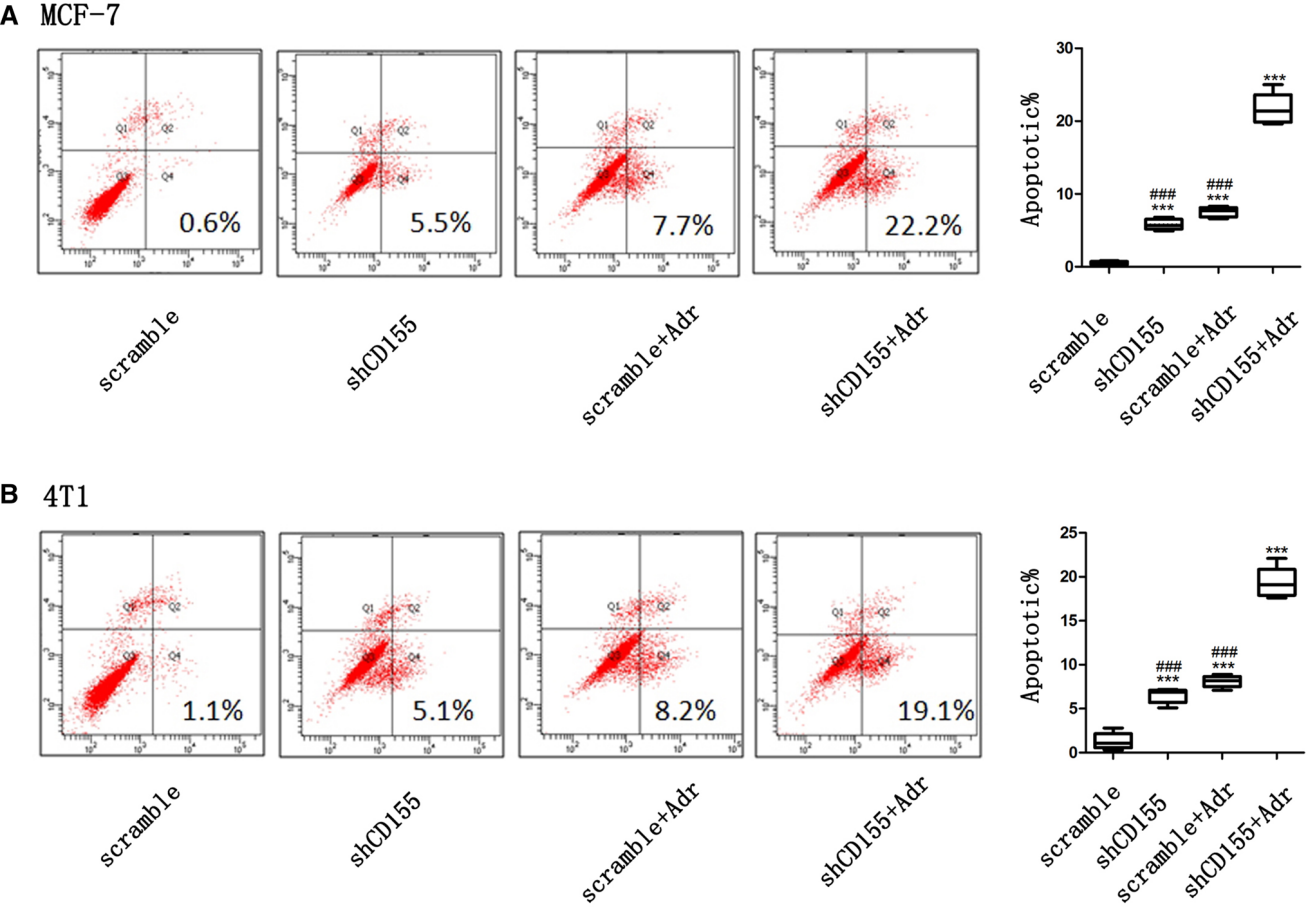
Induction of apoptosis by CD155 downregulation and Adr treatment in vitro. Flowcytometry analyzed apoptosis of breast cancer cells MCF-7 (**a**) and 4T1 (**b**). ***P < 0.001 versus scramble; ^###^*P* < 0.001 versus shCD155 + Adr. Data are expressed as mean ± SD, and all the experiments were performed three times

As CD155 exhibited anti-apoptotic in other cancer cells, the role of CD155 in breast cancer cell apoptosis was investigated. Flowcytometry analysis showed that CD155 knockdown by CD155 shRNA transfection induced apoptosis of both MCF-7 and 4T1 cells, indicating CD155 functions as an anti-apoptotic factor in breast cancer (Fig. 3a, b). Moreover, treatment with Adr also induced apoptosis of these cells (Fig. 3a, b). Importantly, a combination of CD155 knockdown with Adr treatment induced cell apoptosis far more than either of them, indicating that CD155 downregulation synergizes with Adr to induce breast cancer cell apoptosis (Fig. 3a, b).

### Adr induces CD155 expression in breast cancer xenografts

**Fig. 4:**
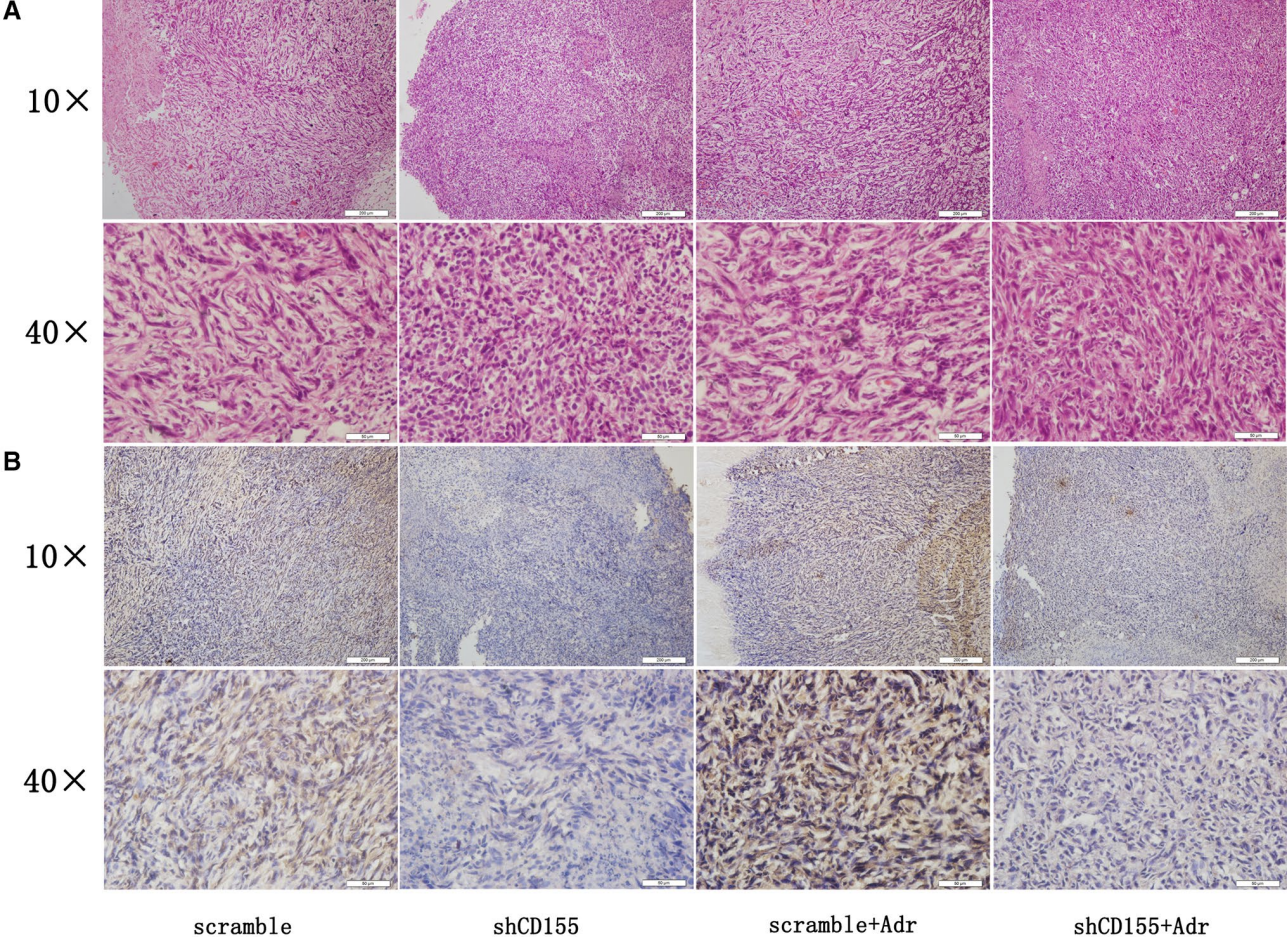
Induction of CD155 expression by Adr treatment in 4T1 allografts. **a** HE staining confirmed the tumor tissues. **b** Immunohistochemistry staining detected CD155 expression in 4T1 xenografts

As 4T1 tumor cells are BALB/c background, we inoculated 4T1 cells with or without CD155 shRNA transfection into BALB/c mice to generate breast cancer xenografts. HE staining confirmed the tumor tissues harvested from these mice (Fig. 4a). As shown by immunohistochemistry staining, CD155 expression was absent in tumors of 4T1 cells transfected with CD155 shRNA, even after these tumors were treated with Adr, however, CD155 expression was upregulated by Adr treatment in tumors of 4T1 cells transfected with scramble (Fig. 4b). These observations indicate that Adr treatment induces CD155 expression in vivo, which can be blocked by CD155 shRNA transfection.

### CD155 downregulation synergizes with Adr to induce cell apoptosis and inhibit the growth of breast cancer xenografts

**Fig. 5:**
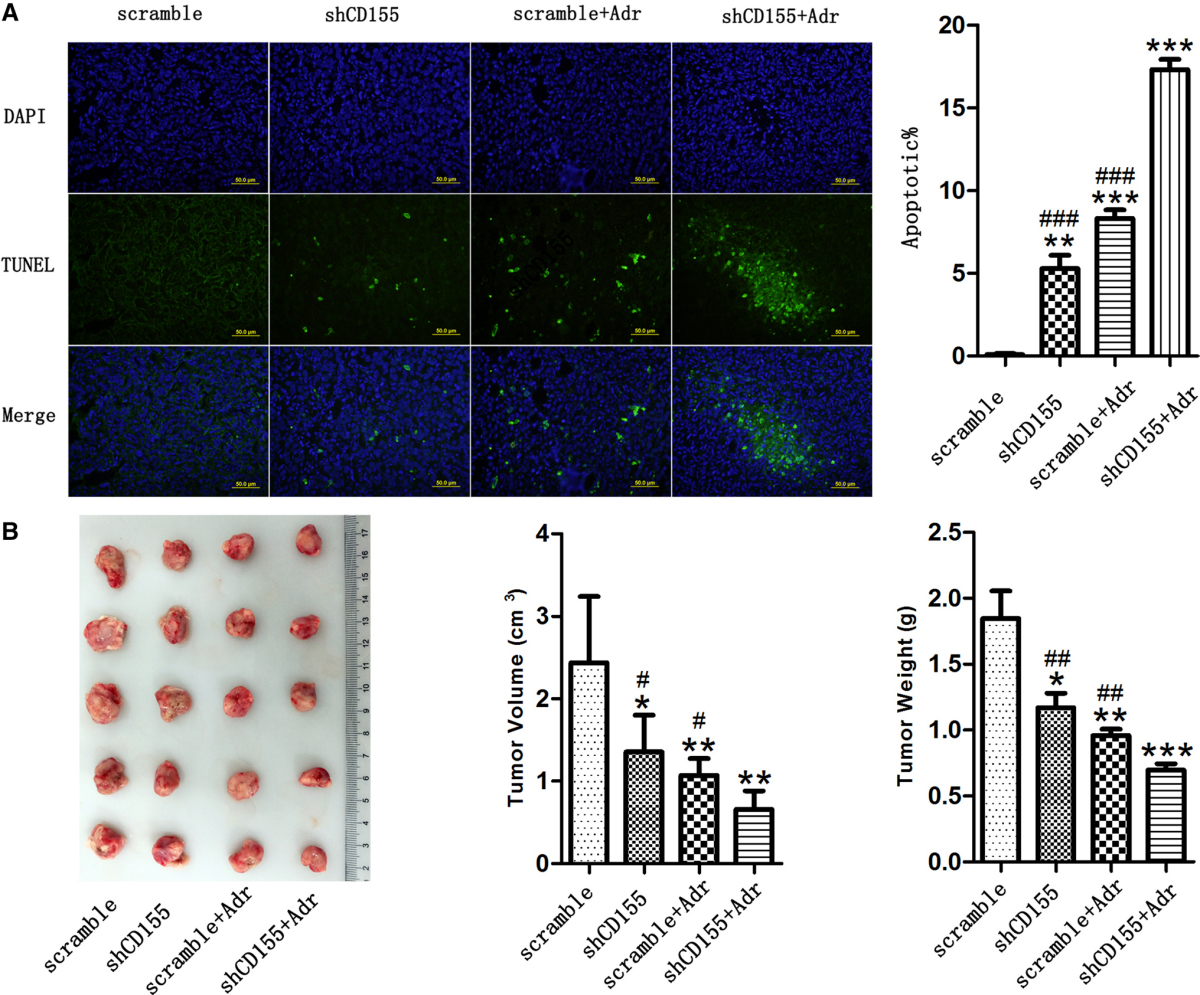
Induction of apoptosis and inhibition of growth by CD155 downregulation and Adr treatment in vivo. **a** TUNEL staining detected cell apoptosis in 4T1 xenografts. ***P < 0.001 versus scramble; ^###^*P* < 0.001 versus shCD155 + Adr. Data are expressed as mean ± SD, and all the experiments were performed three times. **b** Tumor growth was evaluated by measurement of tumor volume and weight. *P < 0.05, **P < 0.01 versus scramble; ^#^*P* < 0.05, ^##^*P* < 0.01 versus shCD155 + Adr. Data are expressed as mean ± SD. Each group contains five mice

TUNEL staining was used to evaluate cell apoptosis in vivo. As shown in Fig. 5a, CD155 knockdown or treatment with Adr significantly increased the apoptotic cells in 4T1 xenografts; and consistent with the in vitro result, the combination of CD155 knockdown with Adr treatment induced more cell death than either of them. Moreover, the growth of 4T1 xenografts was also evaluated by measurement of tumor volume and weight. It was shown that tumor growth was suppressed by CD155 knockdown or Adr treatment, and more significantly by the combination of them (Fig. 5b).

## Discussion

Adr (doxorubicin) is one of the most common chemotherapeutic drugs used to treat cancers including breast cancer. By inhibiting topoisomerase and intercalating itself into the DNA double helix, Adr ceases DNA replication and RNA transcription, and induces cell apoptosis [24]. Noticeably, Adr induces the generation of free radical, leading to further DNA damage [25]. Several signaling molecules such as AMPK, JNK and p53 are involved in Adr-induced cell apoptosis [24]. Moreover, Adr also affects Bcl-2/Bax pathway. It was reported that Adr treatment reduced the expression of anti-apoptotic Bcl-2 whereas increased the expression of pro-apoptotic Bax in breast cancer cells [26].

DNA damage has been shown to induce CD155 expression. Activation of Ataxia telangiectasia mutated (ATM) and ATM- and Rad3 (ATR)-related protein kinases is implicated in CD155 upregulation in tumor cells. Several chemotherapeutic agents such as Adr, melphalan, and bortezomib were shown to induce CD155 expression on multiple myeloma cells, which was blocked by treatment with pharmacologic inhibitors of ATM/ATR-related kinases [19]. Moreover, nitric oxide donors were also found to stimulate CD155 expression on multiple myeloma cells through activation of ATM/ATR-related kinases [18]. Virus infection has been demonstrated to induce DDR, leading to genomic instability and carcinogenesis [27, 28]. Until now it is unclear whether CD155 can be upregulated by tumor-related viruses. However, HIV-1 Vpr protein was reported to upregulate CD155 expression on CD4^+^T lymphocytes through DDR [29].

In the present study, we revealed that Adr treatment upregulated CD155 expression on several breast cancer cells. Furthermore, we showed that CD155 knockdown by shRNA transfection induced breast cancer apoptosis, indicating that endogenous CD155 plays an anti-apoptotic role in these tumor cells. This result is consistent with our previous finding that CD155 knockdown promoted colon cancer cell apoptosis involving downregulation of Bcl-2 and upregulation of Bax [14]. These observations raise a question whether CD155 upregulation by Adr treatment will counteract Adr-induced cell apoptosis.

Apoptosis inhibition is one of the mechanisms underlying chemotherapeutic drug resistance. For instance, upregulation of anti-apoptotic Bcl-2 and downregulation of pro-apoptotic Bax in tumor cells are related to the increased resistance to chemotherapy [30]. Moreover, functional loss of P53 inhibits the apoptotic response and leads to tumor cell senescence and subsequent resistance to chemotherapeutic agents [31]. In the present study, we observed that CD155 downregulation synergized with Adr to induce breast cancer apoptosis in vitro and in vivo, furthermore, CD155 downregulation synergized with Adr to inhibit tumor growth. This observation in combination with our previous finding that CD155 functions as an anti-apoptotic factor by affecting Bcl-2/Bax pathway, suggest that Adr-induced CD155 upregulation is implicated in Adr resistance.

In summary, Adr treatment induces cell apoptosis on one hand, and upregulates CD155 expression on the other hand. CD155 functions as an anti-apoptotic factor which may counteract Adr-induced cell apoptosis, suggesting CD155 induction may be a mechanism underlying Adr resistance by breast cancer cells. Moreover, CD155 has been shown to stimulate cell migration and invasion. Therefore, the effect of Adr-induced CD155 on tumor cell motility needs to be further determined. Taken together, our results demonstrate that targeting CD155 may be potentially used in combination with Adr treatment for breast cancer.

## References

[1] Mendelsohn CL, Wimmer E, Racaniello VR (1989). Cellular receptor for poliovirus: molecular cloning, nucleotide sequence, and expression of a new member of the immunoglobulin superfamily. Cell.

[2] Ikeda W, Kakunaga S, Itoh S, Shingai T, Takekuni K, Satoh K, Inoue Y, Hamaguchi A, Morimoto K, Takeuchi M, Imai T, Takai Y (2003). Tage4/nectin-like molecule-5 heterophilically trans-interacts with cell adhesion molecule nectin-3 and enhances cell migration. J Biol Chem.

[3] Mueller S, Wimmer E (2003). Recruitment of nectin-3 to cell-cell junctions through trans-heterophilic interaction with CD155, a vitronectin and poliovirus receptor that localizes to alpha(v)beta3 integrin-containing membrane microdomains. J Biol Chem.

[4] Ikeda W, Kakunaga S, Takekuni K, Shingai T, Satoh K, Morimoto K, Takeuchi M, Imai T, Takai Y (2004). Nectin-like molecule-5/Tage4 enhances cell migration in an integrin-dependent, nectin-3-independent manner. J Biol Chem.

[5] Kakunaga S, Ikeda W, Shingai T, Fujito T, Yamada A, Minami Y, Imai T, Takai Y (2004). Enhancement of serum- and platelet-derived growth factor-induced cell proliferation by Necl-5/Tage4/poliovirus receptor/CD155 through the Ras-Raf-MEK-ERK signaling. J Biol Chem.

[6] Gao J, Zheng Q, Xin N, Wang W, Zhao C (2017). CD155, an onco-immunologic molecule in human tumors. Cancer Sci.

[7] Masson D, Jarry A, Baury B, Blanchardie P, Laboisse C, Lustenberger P, Denis MG (2001). Overexpression of the CD155 gene in human colorectal carcinoma. Gut.

[8] Nakai R, Maniwa Y, Tanaka Y, Nishio W, Yoshimura M, Okita Y, Ohbayashi C, Satoh N, Ogita H, Takai Y, Hayashi Y (2010). Overexpression of Necl-5 correlates with unfavorable prognosis in patients with lung adenocarcinoma. Cancer Sci.

[9] Nishiwada S, Sho M, Yasuda S, Shimada K, Yamato I, Akahori T, Kinoshita S, Nagai M, Konishi N, Nakajima Y (2015). Clinical significance of CD155 expression in human pancreatic cancer. Anticancer Res.

[10] Iguchi-Manaka A, Okumura G, Kojima H, Cho Y, Hirochika R, Bando H, Sato T, Yoshikawa H, Hara H, Shibuya A, Shibuya K (2016). Increased soluble CD155 in the serum of cancer patients. PLoS ONE.

[11] Sloan KE, Eustace BK, Stewart JK, Zehetmeier C, Torella C, Simeone M, Roy JE, Unger C, Louis DN, Ilag LL, Jay DG (2004). CD155/PVR plays a key role in cell motility during tumor cell invasion and migration. BMC Cancer.

[12] Morimoto K, Satoh-Yamaguchi K, Hamaguchi A, Inoue Y, Takeuchi M, Okada M, Ikeda W, Takai Y, Imai T (2008). Interaction of cancer cells with platelets mediated by Necl-5/poliovirus receptor enhances cancer cell metastasis to the lungs. Oncogene.

[13] Sloan KE, Stewart JK, Treloar AF, Matthews RT, Jay DG (2005). CD155/PVR enhances glioma cell dispersal by regulating adhesion signaling and focal adhesion dynamics. Cancer Res.

[14] Zheng Q, Wang B, Gao J, Xin N, Wang W, Song X, Shao Y, Zhao C (2018). CD155 knockdown promotes apoptosis via AKT/Bcl-2/Bax in colon cancer cells. J Cell Mol Med.

[15] Solecki DJ, Gromeier M, Mueller S, Bernhardt G, Wimmer E (2002). Expression of the human poliovirus receptor/CD155 gene is activated by sonic hedgehog. J Biol Chem.

[16] Kamran N, Takai Y, Miyoshi J, Biswas SK, Wong JS, Gasser S (2013). Toll-like receptor ligands induce expression of the costimulatory molecule CD155 on antigen-presenting cells. PLoS ONE.

[17] Ardolino M, Zingoni A, Cerboni C, Cecere F, Soriani A, Iannitto ML, Santoni A (2011). DNAM-1 ligand expression on Ag-stimulated T lymphocytes is mediated by ROS-dependent activation of DNA-damage response: relevance for NK-T cell interaction. Blood.

[18] Fionda C, Abruzzese MP, Zingoni A, Soriani A, Ricci B, Molfetta R, Paolini R, Santoni A, Cippitelli M (2015). Nitric oxide donors increase PVR/CD155 DNAM-1 ligand expression in multiple myeloma cells: role of DNA damage response activation. BMC Cancer.

[19] Soriani A, Zingoni A, Cerboni C, Iannitto ML, Ricciardi MR, Di GV, Cippitelli M, Fionda C, Petrucci MT, Guarini A, Foà R, Santoni A (2009). ATM-ATR-dependent up-regulation of DNAM-1 and NKG2D ligands on multiple myeloma cells by therapeutic agents results in enhanced NK-cell susceptibility and is associated with a senescent phenotype. Blood.

[20] Carvalho C, Santos RX, Cardoso S, Correia S, Oliveira PJ, Santos MS, Moreira PI (2009). Doxorubicin: the good, the bad and the ugly effect. Curr Med Chem.

[21] Rivankar S (2014). An overview of doxorubicin formulations in cancer therapy. J Cancer Res Ther.

[22] StatPearls (2018). StatPearls.

[23] Bailey-Downs LC, Thorpe JE, Disch BC, Bastian A, Hauser PJ, Farasyn T, Berry WL, Hurst RE, Ihnat MA (2014). Development and characterization of a preclinical model of breast cancer lung micrometastatic to macrometastatic progression. PLoS ONE.

[24] Tacar O, Dass CR (2013). Doxorubicin-induced death in tumour cells and cardiomyocytes: is autophagy the key to improving future clinical outcomes. J Pharm Pharmacol.

[25] Minotti G, Menna P, Salvatorelli E, Cairo G, Gianni L (2004). Anthracyclines: molecular advances and pharmacologic developments in antitumor activity and cardiotoxicity. Pharmacol Rev.

[26] Leung LK, Wang TT (1999). Differential effects of chemotherapeutic agents on the Bcl-2/Bax apoptosis pathway in human breast cancer cell line MCF-7. Breast Cancer Res Treat.

[27] Cerboni C, Fionda C, Soriani A, Zingoni A, Doria M, Cippitelli M, Santoni A (2014). The DNA damage response: a common pathway in the regulation of NKG2D and DNAM-1 ligand expression in normal, infected, and cancer cells. Front Immunol.

[28] Hollingworth R, Grand RJ (2015). Modulation of DNA damage and repair pathways by human tumour viruses. Viruses.

[29] Vassena L, Giuliani E, Matusali G, Cohen ÉA, Doria M (2013). The human immunodeficiency virus type 1 Vpr protein upregulates PVR via activation of the ATR-mediated DNA damage response pathway. J Gen Virol.

[30] Mansoori B, Mohammadi A, Davudian S, Shirjang S, Baradaran B (2017). The different mechanisms of cancer drug resistance: a brief review. Adv Pharm Bull.

[31] Bertheau P, Lehmann-Che J, Varna M, Dumay A, Poirot B, Porcher R, Turpin E, Plassa LF, de Roquancourt A, Bourstyn E, de Cremoux P, Janin A, Giacchetti S, Espié M, de Thé H (2013). p53 in breast cancer subtypes and new insights into response to chemotherapy. Breast.

